# The complete mitochondrial genome of *Notonecta chinensis* Fallou and implications for the phylogeny of Notonectidae (Hemiptera: Heteroptera)

**DOI:** 10.1080/23802359.2017.1413303

**Published:** 2017-12-08

**Authors:** Min Li, Danli Zhang, Wanqing Zhao, Hufang Zhang, Shengcai Li, Teng Li

**Affiliations:** a College of Agriculture, Shanxi Agricultural University, Taigu, China;; b Department of Biology, Taiyuan Normal University, Taiyuan, China;; c Department of Biology, Xinzhou Teachers University, Xinzhou, China;; d Institute of Zoology and Developmental Biology, College of Life Sciences, Lanzhou University, Lanzhou, China

**Keywords:** Mitochondrial genome, *Notonecta chinensis*, Nepomorpha, phylogeny

## Abstract

The complete mitochondrial genome of *Notonecta chinensis* Fallou was sequenced and was 15,149 bp long with 75.6% A + T. There were 37 typical genes including 13 protein-coding genes (PCGs), 22 transfer RNA (tRNAs), and two ribosomal RNA (rRNAs). The 531 bp D-loop region was located between 12S rRNA and trnI-*Ile*. All PCGs started with ATN codons, except COI and ND4L, and ended with TAA, except COII, COIII, ATPase6 and ND3. Phylogenetic analyses highly supported the monophyly for each superfamily. Notonectoidea was formed a solid monophyletic group, with *N. chinensis* and *Enithares tibialis* clustered into one clade.

The backswimmers, Notonectidae (Hemiptera: Heteroptera: h and Slater 1995). They are predaceous insects and some species are natural enemies and potential biocontrol agents against *Anopheles dirus, Anopheles sinensis, Culex pipiens, Culex quinquefasciatus* and *Aedes togoi* (Ellis and Borden 1970; Sana et al. 2008; Fischer et al. 2012). In this study, the mt-genome of h and Slater 1995). They are predaceous insects and some species are natural enemies and potential biocontrol agents against *Anopheles dirus, Anopheles sinensis, Culex pipiens, Culex quinquefasciatus* and *Aedes togoi* (Ellis and Borden 1970; Sana et al. 2008; Fischer et al. 2012). In this study, the mt-genome of *Notonecta chinensis* which collected from Ningyang County, Taian City, Shandong Province, China, on 24 August 2008 was sequenced and analyzed.

The complete mt-genome of *N. chinensis* was a typically circular molecule with 15,149 bp in length (GenBank accession no. KJ584365), and consists of 13 protein coding genes (PCGs), 22 tRNA genes, two rRNA genes and a control region. The nucleotide composition of *N. chinensis* mt-genome was significantly biased toward A and T, with an A + T content of 75.6% (A = 42.9%, T = 32.7%, C = 14.0%, G = 10.4%). The nucleotide skew statistics for the whole mt-genome and J-strand PCGs were AT-skewed and CG-skewed, whereas the N-strand PCGs were GC-skewed and much more TA-skewed.

Gene overlaps were observed at 13 gene junctions and involved a total of 41 bp, while the longest overlap (8 bp) existed between tRNA-Trp and tRNA-Cys. The two overlaps between two gene pairs ATP8/ATP6 and ND4L/ND4 are the same seven nucleotides (ATGATAA). Eleven of the thirteen PCGs of *N. chinensis* initiated with ATN as start codon (seven with ATG, three with ATA and one with ATT). COI gene used a non-traditional start codon TTG, and ND4L gene started with unusual codon GTG. Most PCGs stopped with the complete termination codon (seven with TAA and two with TAG), whereas the remaining four PCGs were ended with an incomplete stop codon T.

The mt-genome of *N. chinensis* was employed to reconstruct the phylogenetic trees of 13 PCGs with the following 14 mt-genomes of closely related insects from GenBank. GPU MrBayes (Bao et al. 2013) was employed to reconstruct the phylogenetic tree with the GTR + I + G model estimated by Modeltest Version 3.7 (Posada and Crandall 1998). Each superfamily formed a monophyletic cluster with a high degree of bootstrap support, and Notonectiodea was monophyletic. Notonectiodea was more closely related to Pleoidea, and *N. chinensis* and *Enithares tibialis* were sibling clusters (Figure 1) The complete mitogenome of *N. chinensis* provides important molecular and evolutionary evidence for *Notonecta* and Notonectidae.

**Figure 1.**
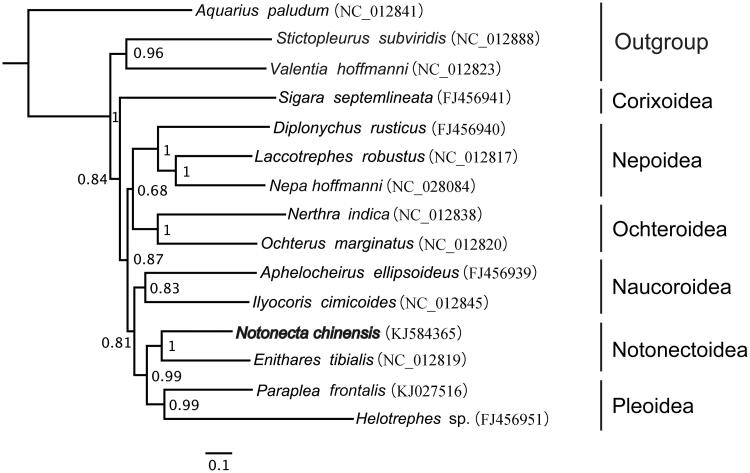
Phylogenetic relationship of *Notonecta chinensis* within Nepomorpha inferred from 13 PCGs. Numbers at the nodes are bootstrap values.

## References

[CIT0001] Bao J, Xia HJ, Zhou JF, Liu XG, Wang G. 2013 Efficient implementation of MrBayes on multi-GPU. Mol Biol Evol. 30:1471–1479. 23493260 10.1093/molbev/mst043 PMC3649675

[CIT0002] Ellis RA, Borden JH. 1970 Predation by *Notonecta undulata* (Heteroptera: Notonectidae) on larvae of the yellow-fever mosquito. Ann Entomol Soc Am. 63:963–973.

[CIT0003] Fischer S, Pereyra D, Fernández L. 2012 Predation ability and non-consumptive effects of *Notonecta sellata* (Heteroptera: Notonectidae) on immature stages of *Culex pipiens* (Diptera: Culicidae). J Vector Ecol. 37:245–250. 22548560 10.1111/j.1948-7134.2012.00223.x

[CIT0004] Posada D, Crandall KA. 1998 Modeltest: testing the model of DNA substitution. Bioinformatics. 14:817–818. 9918953 10.1093/bioinformatics/14.9.817

[CIT0005] Sana N, Aditya G, Bal A, Saha GK. 2008 Influence of light and habitat on predation of *Culex quinquefasciatus*, (Diptera: Culicidae) larvae by the waterbugs (Hemiptera: Heteroptera). Insect Sci. 15:461–469.

[CIT0006] Schuh RT, Slater JA. 1995 True bugs of the world (Hemiptera: Heteroptera): classification and natural history. Ithaca: Cornell University Press, xii + 338 p.

